# Infantile onset Sandhoff disease: clinical manifestation and a novel common mutation in Thai patients

**DOI:** 10.1186/s12887-020-02481-3

**Published:** 2021-01-07

**Authors:** Thipwimol Tim-Aroon, Khunton Wichajarn, Kamornwan Katanyuwong, Pranoot Tanpaiboon, Nithiwat Vatanavicharn, Kullasate Sakpichaisakul, Arthaporn Kongkrapan, Jakris Eu-ahsunthornwattana, Supranee Thongpradit, Kanya Moolsuwan, Nusara Satproedprai, Surakameth Mahasirimongkol, Tassanee Lerksuthirat, Bhoom Suktitipat, Natini Jinawath, Duangrurdee Wattanasirichaigoon

**Affiliations:** 1 Division of Medical Genetics, Department of Pediatrics, Faculty of Medicine Ramathibodi Hospital, Mahidol University, Bangkok, Thailand; 2 Department of Pediatrics, Faculty of Medicine, Khon Kaen University, Khon Kaen, Thailand; 3 Department of Pediatrics, Faculty of Medicine, Chiang Mai University, Chiang Mai, Thailand; 4 Children’s National Rare Disease Institute, Children’s National Hospital, Washington, DC USA; 5 Division of Medical Genetics, Department of Pediatrics, Faculty of Medicine Siriraj Hospital, Mahidol University, Bangkok, Thailand; 6 Division of Neurology, Department of Pediatrics, Queen Sirikit National Institute of Child Health, Ministry of Public Health, Bangkok, Thailand; 7 College of Medicine, Rangsit University, Bangkok, Thailand; 8 Department of Community Medicine, Faculty of Medicine Ramathibodi Hospital, Mahidol University, Bangkok, Thailand; 9 Research Center, Faculty of Medicine Ramathibodi Hospital, Mahidol University, Bangkok, Thailand; 10 Program in Translational Medicine, Faculty of Medicine Ramathibodi Hospital, Mahidol University, Bangkok, Thailand; 11 Department of Medical Science, Ministry of Public Health, Nonthaburi, Thailand; 12 Department of Biochemistry, Faculty of Medicine Siriraj Hospital, Mahidol University, Bangkok, Thailand; 13 Integrative Computational Bioscience Center, Mahidol University, Salaya, Nakhon Pathom, Thailand

**Keywords:** GM2 gangliosidosis, Sandhoff disease, *HEXB*, Tay-Sachs disease, Developmental regression, Neurometabolic disorder, Thai

## Abstract

**Background:**

Sandhoff disease (SD) is an autosomal recessive lysosomal storage disorder, resulting in accumulation of GM2 ganglioside, particular in neuronal cells. The disorder is caused by deficiency of β-hexosaminidase B (HEX-B), due to pathogenic variant of human *HEXB* gene.

**Method:**

This study describes clinical features, biochemical, and genetic defects among Thai patients with infantile SD during 2008–2019.

**Results:**

Five unrelated Thai patients presenting with developmental regression, axial hypotonia, seizures, exaggerated startle response to noise, and macular cherry red spot were confirmed to have infantile SD based on deficient HEX enzyme activities and biallelic variants of the *HEXB* gene. In addition, an uncommon presenting feature, cardiac defect, was observed in one patient. All the patients died in their early childhood. Plasma total HEX and HEX-B activities were severely deficient. Sequencing analysis of *HEXB* gene identified two variants including c.1652G>A (p.Cys551Tyr) and a novel variant of c.761T>C (p.Leu254Ser), in 90 and 10% of the mutant alleles found, respectively. The results from in silico analysis using multiple bioinformatics tools were in agreement that the p.Cys551Tyr and the p.Leu254Ser are likely pathogenic variants. Molecular modelling suggested that the Cys551Tyr disrupt disulfide bond, leading to protein destabilization while the Leu254Ser resulted in change of secondary structure from helix to coil and disturbing conformation of the active site of the enzyme. Genome-wide SNP array analysis showed no significant relatedness between the five affected individuals. These two variants were not present in control individuals. The prevalence of infantile SD in Thai population is estimated 1 in 1,458,521 and carrier frequency at 1 in 604.

**Conclusion:**

The study suggests that SD likely represents the most common subtype of rare infantile GM2 gangliosidosis identified among Thai patients. We firstly described a potential common variant in *HEXB* in Thai patients with infantile onset SD. The data can aid a rapid molecular confirmation of infantile SD starting with the hotspot variant and the use of expanded carrier testing.

**Supplementary Information:**

The online version contains supplementary material available at 10.1186/s12887-020-02481-3.

## Background

GM2 gangliosidoses including Tay-Sachs disease (TSD, MIM 272800), Sandhoff disease (SD, MIM 268800), and GM2 activator protein deficiency (GM2AP or AB variant, MIM 272750) are a group of lysosomal storage disorders of which the breakdown of glycosphingolipids, GM2 gangliosides, is impaired, leading to an accumulation of the substrates in various internal organs, particular in neuronal cells [1]. These disorders are resulted from deficiency of β-hexosaminidase enzyme (HEX), or rarely, defect of activator protein. Two isoforms of HEX includes β-hexosaminidase A (HEX-A) consisting of a heterodimer between α- and β-subunit (αβ), and β-hexosaminidase B (HEX-B) consisting of a homodimer of β-subunit (ββ) [2]. TSD, SD, and GM2AP deficiency are caused by biallelic pathogenic variants of human *HEXA, HEXB, GM2AP* gene in respective order [1]. *HEXB* is located on 5q13, containing 14 coding exons and spanning mRNA of 2 kb, and encoding 556 amino acids.

There are three clinical subtypes in TSD and SD including infantile, juvenile, and adult subtypes [1]. Infantile manifestations of TSD, SD, and GM2AP are clinically indistinguishable. The infantile disease is characterized by development of axial hypotonia, startle response with hyperacusis, developmental regression starting at the age of 3–6 months, followed by progressive loss of motor skill, neurologic signs of upper and lower motor neurons, macrocephaly, seizures, macular cherry-red spots, and eventually death before 3–5 years of age [1, 3, 4].

Patients with juvenile and adult forms present in early-late childhood or adulthood with slow progression of neurological symptoms such as developmental delay, abnormal gait, ataxia, spinocerebellar and motor neuron dysfunction, increased myotatic reflexes which sometimes are misdiagnosed with late-onset spinal muscular atrophy, and Friedreich ataxia [1, 5].

The diagnosis of SD can be confirmed by biochemical findings of low total HEX and deficient HEX-B activities, with high percentage of HEX-A/total HEX activity.

The prevalence of GM2 gangliosidosis in Thailand is not known. Only two cases of SD without molecular data [6, 7] and a single patient with TSD [8] were previously described. During a 12-year period of 2008–2019, our laboratory is only one center in Thailand to provide biochemical analysis for GM2 gangliosidoses, of which 5 out of 8 specimens were confirmed to have SD and none for TSD. Herein, we describe clinical, biochemical, and molecular characteristics of the five patients with infantile-onset SD and *HEXB* variants found among the Thai patients.

## Methods

### Patients

Five unrelated patients (2 female and 3 male) biochemically confirmed of having SD were included in the study. Genetic analysis was performed following the approval of Ramathibodi Hospital Institutional Review Board.

### Biochemical analysis

Measurement of the patients’ plasma total HEX and HEX-B enzyme activity was determined using fluorogenic substrate (0.95 mg/ml) 4-methylumbelliferyl-2-acetomido-2-deoxy-β-D-glucopyranoside (4MUG, Sigma, MO) with differential heat inactivation, following established protocol [9]. Briefly, total HEX activity was estimated by incubation at 37 °C for 20 min in the presence of 2.5 mM 4MUG and 0.01 M citrate-phosphate buffer, pH 4.4 in a total volume of 0.25 ml [9]. Heat inactivation was processed by pre-incubating samples for 180 min at 52 °C before adding 4MUG and incubation at 37 °C for 20 min; after which reactions were stopped by adding 1.25 ml of 0.17 M glycine-carbonate buffer, pH 9.8. This process led to loss of HEX-A, which is heat-labile [9]. Therefore, HEX-B was determined as the activity at this point. Fluorescence excitation was processed at 365 nm wavelength and emission was determined at 450 nm. The %HEX-A activities were calculated by subtracting HEX-B activity from total HEX activity. All samples were performed in triplicate.

### Genetic analysis

Genomic DNA was extracted from peripheral blood specimens following standard protocols (QIAGEN GmbH). PCR primers for each of the coding exons of *HEXB* gene and its intron-flanking sequences were designed using program PRIMER 3 (http://www.frodo.wi.mit.edu/cgi-bin/primer3). GenBank reference sequences were NT_006713, NM_000521 and NP_000512. PCR and direct sequencing of all the exons were performed, primer sequences and PCR conditions were provided as supplemental data (Table S1).

Once a variant(s) was identified, specimens from parents and available sibs were tested for the variant discovered. For screening of variants identified, 50 healthy control individuals (regular blood donors) were analyzed using restriction digest with appropriate endonuclease restriction enzyme(s). Later, data from an in-house (unpublished) whole exome database of 496 Thai individuals affected with other known/unknown disorders but not related to the SD-related phenotypes was checked for the presence of the mutant allele. To predict the effect of missense variant, we performed in silico analysis using multiple software tools. Multiple protein sequence alignment of vertebrate species was performed by using Clustal Omega software (https://www.ebi.ac.uk/Tools/msa/clustalo).

### Prediction of the structure and function of the mutant protein

To predict the structural and functional changes of the HEXB mutant protein, I-TASSER, COTH and Mutation Taster were used for analysis (reference sequence NP_000512.1) [10–12]. The 3D homological structures of mutant protein were constructed using SWISS-MODEL [13]. The HEXB with PDB-ID: 1o7a was choose as the template [14]. The model was visualized, compared and analyzed using UCSF chimera [15].

### Detection of common founders

To identify common ancestry among the patients with the rare variant detected, we performed whole genome genotyping using Infinium Asian Screening Array version 1.0. All five probands were genotyped including 19 randomly selected unrelated control from Thai population.

A total of 35,725 SNPs on chromosome 5 were converted to forward strand base and non-polymorphic variants were removed, leaving 25,624 SNPs for subsequent analysis.

Phasing was performed using Eagle version 2 [16] with the non-European or mixed population from the Haplotype Reference Consortium (HRC Version r1.1 2016) [17] as a reference population. Phasing was carried out on the Michigan Imputation Server [18].

To detect a shared identity-by-descent/identity-by-state (IBD/IBS) indicating shared founders among cases, we used GERMLINE version 1.5.3 [19] specifying the option -min_m 1 and -bits 50 aiming to detect a minimum size of 1 cM IBD segment. A total of 18,733 polymorphic SNPs with minor allele frequency (MAF) > 0.05 were included in this part of the analysis. Subsequently, ERSA version 2.1 was used to test for a statistically significant shared recent common ancestry [20].

## Results

### Clinical characteristics

**Fig. 1:**
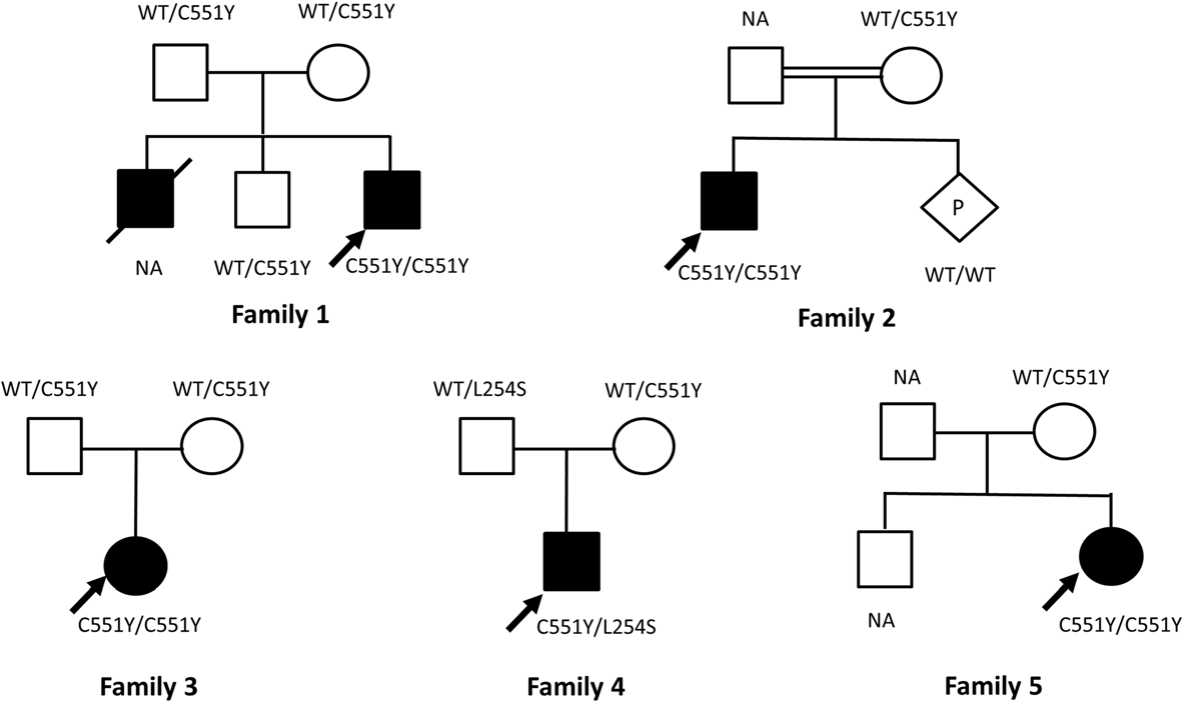
Pedigrees of 5 affected families. Noted their genotypes, black square/circle indicating affected individual

All patients were unrelated and lived in different provinces located in the Central (1), Northern (1) and Northeastern (3) Thailand. The mean age of disease onset was 6.6 months and the median age of diagnosis was 17 months (range 16–34 months) (Table 1). The mean duration of time to diagnosis was 15 months (range 11–26 months). Developmental regression is the first manifestation in all patients. Despite normal psychomotor development during the first 6 months of life, patient-1 had been misdiagnosed of having cerebral palsy. At the age of 9 months, patient-1 was incidentally heard to have heart murmur and later confirmed to have mild mitral valve prolapse and moderate mitral regurgitation [6]. Macular cherry red spot and excessive startle response to noise were present in all patients. Macrocephaly was noted in two, and seizures in all of the patients. Myoclonic seizure exacerbated by loud noise was observed in 3 out of 5 patients whereas generalized tonic clonic seizures were also commonly found (Table 1). Mild prematurity was observed in two patients. Four patients were sporadic cases, except for patient-1 who had a deceased elder brother who died due to progressive neurological regression of unclear etiology at the age of 3 years (Fig. 1a). Parental consanguinity was present in family-2.

**Table 1:** Clinical characteristics of five Thai patients with infantile SD

Patient/Sex	Onset age	Dx age	GA/BW	Seizures and onset	Macular cherry red spot	Enlarged liver and spleen	Macrocephaly/ Other findings	Brain imaging	Age at death
1/M^a^	6 mo	17 mo	term/NA	GTC, MCS aggravated by loud noise at 15 mo	yes	no	no/ MVP spasticity, hyperreflexia	Cranial ultrasound: normal at 10 mo	2 yo
2/M^b^	8 mo	25 mo	NA/NA	MCS aggravated by loud noise at 8 mo	yes	no	no/ spasticity, hyperreflexia	MRI: hypomyelination, hyperintensity of bilateral thalamus	4 yo
3/F	5 mo	16 mo	36 wk./ 2360 g	GTC at 12 mo	yes	mild hepato megaly	yes/ NA	MRI: symmetrical homogeneous increased density of bilateral thalamus with subcortical white matter change of bilateral frontal and temporal lobes, mild brain atrophy	2 yo
4/M	6 mo	16 mo	36 wk./ 2560 g	not yet developed at 16 mo	yes	no	no/ axial hypotonia, hyperreflexia	MRI: diffuse hyper T2 signals at bilateral cerebral white matter, and hypo signal intensity of bilateral thalami; thinning of corpus callosum and cerebellar peduncles	6 yo
5/F	8 mo	34 mo	Term/ 3400 g	MCS at 10 mo	yes	no	yes/ axial hypotonia, hyperreflexia	MRI: mild atrophic change of bilateral temporal lobes, mild prominent third ventricle and bilateral lateral ventricles	4 yo

*BW* birth weight, *Dx* diagnosis, *g* grams, *GA* gestational age, *GTC* generalized tonic clonic seizures, *MCS* myoclonic seizures, *mo* months, *MVP* mitral valve prolapse and regurgitation, *NA* not available, *wk.* weeks, *yo* years

^a^ Sakpichaisakul K. et al. J Med Assoc Thai. 2010;93:1088–1092; ^b^ grandparents are full siblings

### Enzyme activities and HEXB variants

All patients have significantly low total HEX activities ranging from 3.0–9.6% of normal control. The HEX-B activities were ranging 0.9–2.7% of normal control, while % of HEX-A/total HEX activities were elevated as expected (Table 2).

**Table 2:** Biochemical and mutation data

Patient	Enzyme assay in plasma		*HEXB* mutation			
	Total HEX (% of normal range)^a^	HEX-B activity (% of normal range)^b^	% HEX-A/ total HEX^c^	Zygosity	Base change	Amino acid change
1	21.9 (3.0%)	3.0 (1.1%)	89.0	homo	c.1652G>A	p.Cys551Tyr
2	70.2 (9.6%)	7.7 (2.7%)	89.0	homo	c.1652G>A	p.Cys551Tyr
3	48.9 (6.7%)	7.6 (2.6%)	84.5	homo	c.1652G>A	p.Cys551Tyr
4	33.1^d^ (4.1%)	NA	95.1^d^	het het	c.1652G>A c.761 T>C	p.Cys551Tyr p.Leu254Ser
5	46.1 (6.3%)	2.6 (0.9%)	94.5	homo	c.1652G>A	p.Cys551Tyr

*het* heterozygous, *homo* homozygous

^a^ Total HEX reference range: 729 ± 225.6 nmol/hr./ml; % of normal range as compared to the according mean reference range

^b^ HEX-B activity reference range: 288.9 ± 59.1 nmol/hr./ml; % of normal range as compared to the according mean reference range

^c^ % HEX-A/total HEX reference range: 59.3 ± 6.3%

^d^ reference ranges for total enzyme activity and % HEX-A/total HEX activity were 801 ± 190 nmol/mg prot/hr. and 55–72%, respectively (National Taiwan University Hospital Laboratory)

**Fig. 2:**
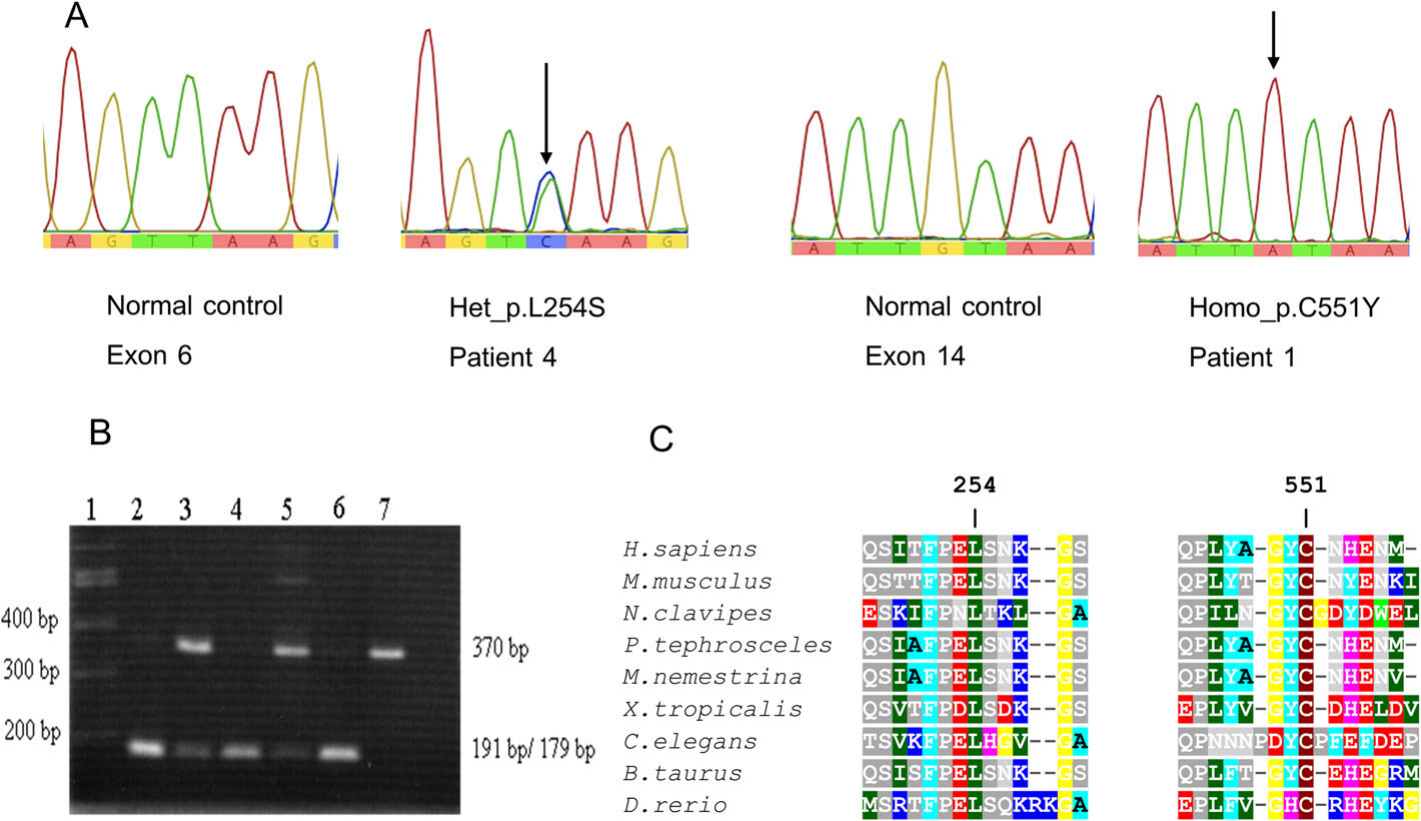
Mutant sequences and protein alignment. **a** Genomic DNA sequences showing *HEXB* variants. Noted heterozygous c.761T>C (p.Leu254Ser) in patient-4, homozygous c.1652G>A (p.Cys551Tyr) in patient-1, and sequence of normal control on side by side. **b** PCR-*PsiI* restriction digest. Noted the c.1652G>A mutant allele creating a *Psil* restriction site, yielding 179 and 191 bp allele in homozygous individuals (patient-1, −2 and − 3 in lanes 2, 4 and 6, respectively) whereas heterozygous individuals harbored the uncut normal allele of 370 bp in addition to the 179 and 191 bp from the mutant allele (unaffected brother of patient-1 and the mother of patient-2 in lanes 3 and 5, respectively). **c** Protein sequence alignment of HEXB across various vertebrate species. Noted highly conserved nature of the Cysteine551 and the Leucine254 residues

A nucleotide substitution, c.1652G>A (p.Cys551Tyr) of the exon 14 was identified in homozygous state in 4 patients and present in compound heterozygous state with another variant (in *trans*), c.761T>C (p.Leu254Ser) of exon 6, in one patient (Fig. 2a). Eight of 10 parents were tested and found to be heterozygous for either p.Cys551Tyr or p.Leu254Ser. The in silico analyses using SIFT, PolyPhen2, PROVEAN, PredictSNP2, CADD, DANN, FATHMM, FunSeq2 and GWAVA were in agreement, suggesting deleterious effect of both missense alleles (supplemental data, Table S2). The c.1652G>A variant created a *Psi*I restriction site which was used as the second method to confirm the presence of this variant in the patients, relatives and controls (Fig. 2b). Comparative in silico analysis of the p.Cys551Tyr of and the p.Leu254Ser showed evolutionary conservation among vertebrate species of Cys551 and Leu254 residues (Fig. 2c). The two variants were not found in our in-house whole exome database and in the gnomAD (allele frequency = 0). The c.1652G>A was assigned as uncertain significance (PM2, PP2, PP3) while the c.761T>C was classified as likely pathogenic (PM2, PP2, PP3, PP5), based on American College of Medical Genetics and Genomics (ACMG) and the Association for Molecular Pathology (AMP) (https://varsome.com/variant/hg19).

### Molecular modelling of the mutant protein

**Fig. 3:**
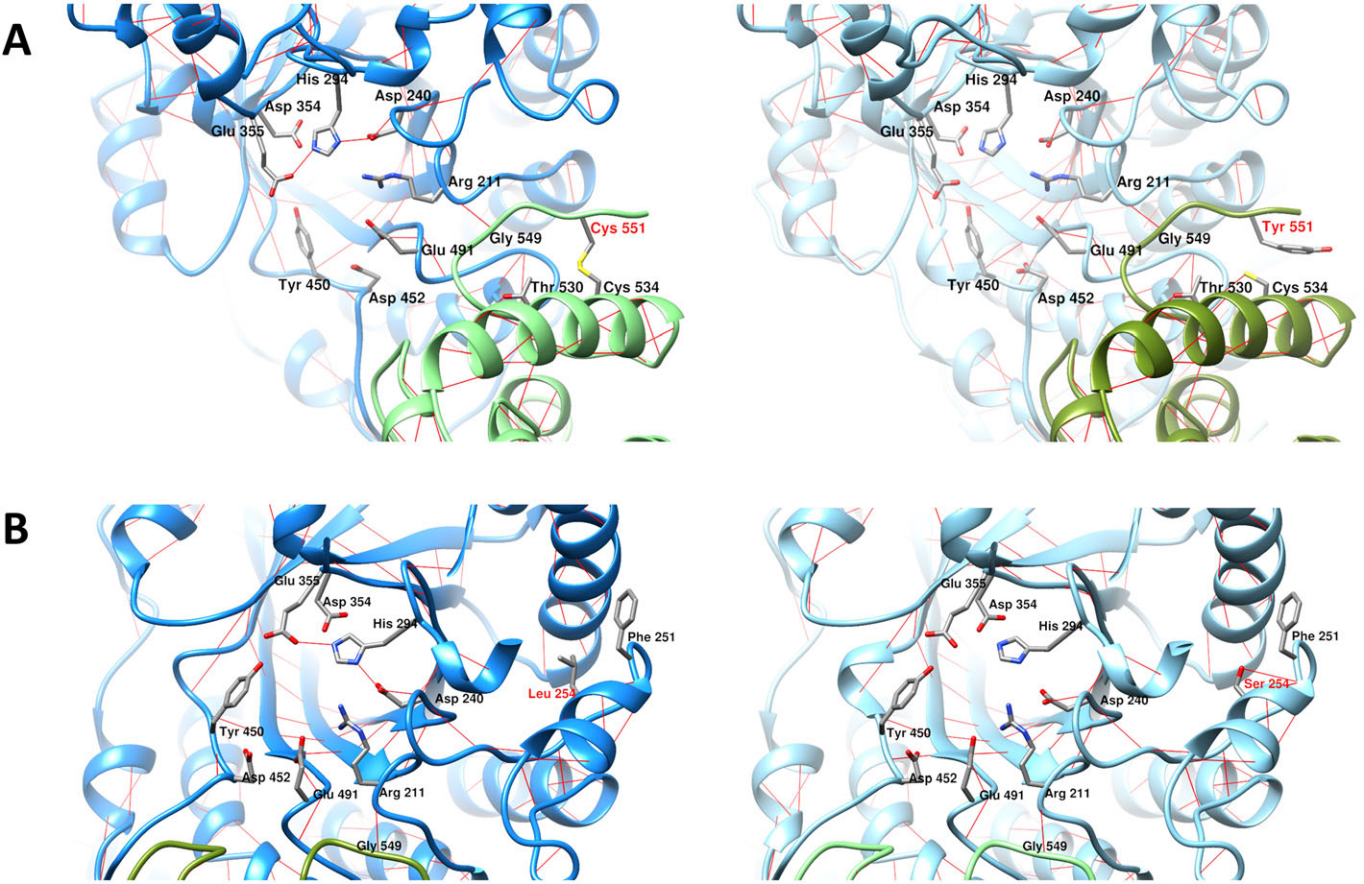
Structural homology of the Cys551Tyr and Leu254Ser. **a** Molecular model of Cys551Tyr. Noted loss of the disulfide bond between Cys534 and Cys551 and additional hydrogen bond formation between Cys534 and Thr530. **b** Homological model of Leu254Ser, indicating one additional hydrogen bond forming between Ser254 and Phe251

The overall features of structural analysis were summarized in Table 3. The homological structures were able to build with qualitative model energy analysis (QMEAN) value indicating that the predicted structure was with good quality [21]. The replacement of Cys551 by Tyr led to the loss of disulfide bond with Cys534 and created a hydrogen bond formation with the carboxyl group of Thr530 as predicted by MutationTaster (Fig. 3a). The variant from Leu254 to Ser254 resulted in altered secondary structure, from helix (amino acid 253–258) to coil as indicated by I-TASSER and MutationTaster. The p.Leu254Ser created one additional hydrogen bond formed between the hydroxyl group of Ser254 and the carboxyl group of Phe251, which is predicted to destroy the helical structure and decrease protein stability (Fig. 3b) [22].

**Table 3:** In silico structural analysis of *HEXB* mutations: Leu254Ser and Cys551Tyr

	Structural analysis			Dimerization capability	
	I-TASSER	SWISS-MODEL (QMEAN)	MutationTaster	SWISS-MODEL (QSQE)	COTH
Leu254	helix	template: 1o7a	–	–	yes
Ser254	coil	−0.77	helix loss (253–258)	1	yes
Cys551	coil	template: 1o7a	–	–	yes
Tyr551	coil	−0.67	disulfide loss (551)	1	yes
Ser254-Tyr551	–	–	–	–	yes

QMEAN, the Qualitative Model Energy Analysis; QSQE, quaternary structure quality estimate

COTH and SWISS-MODEL showed that homodimerization Cys551Tyr-Cys551Tyr could be formed in the case of homozygous state whereas Cys551Tyr-Cys551Tyr, Leu254Ser-Leu254Ser, and Leu254Ser-Cys551Tyr could be found in compound heterozygosity, though the dimers may be less stable in both cases. SWISS-MODEL was able to predict the homodimerization between the two identical subunits, while COTH was more flexible to predict the homodimerization of the non-identical subunits (Table 3). The quaternary structure quality estimate (QSQE) is equal to 1 (range 0–1), reflecting accurate prediction of the oligomerization [23].

### Common founders

GERMLINE identified 1539 segments shared IBS with the median size of 1.43 cM (range from 1 to 4.58). On average, the combined shared segments between each individual was 9.39 cM (SD = 0.519). However, there was no significant relatedness detected between each pair of cases (*p*-value = 1).

## Discussion

We described five patients with infantile onset SD who presented with characteristic features of GM2 gangliosidosis. Pattern of generalized tonic-clonic seizures and myoclonic seizures exacerbated by loud noise was consistent with previous observation [3]. Unlike TSD, mild hepatosplenomegaly can be noted in SD as also found in one patient of the present cohort (patient-3) [24]. Cardiac involvement including cardiomegaly, valvulopathies, hypertrophic/dilated cardiomyopathy is an uncommon finding, it is rare but could be a presenting feature of infantile SD and even ahead of neurological manifestation [25, 26].

Late stage SD could be misdiagnosed as cerebral palsy and the cause of seizures was though due to cerebral palsy and vice versa, as seen in one of our patients. A careful history taking including detailed progression of psychomotor retardation and seizures pattern is essential to alert pediatricians to this rare disorder.

For the first time, we described molecular characteristics of infantile SD in Southeast Asian descendants which is an underrepresented population. It is surprising that most of the Thai patients shared a common allele, c.1652G>A (p.Cys551Tyr). The mutant p.Cys551Tyr is expected to cause protein destabilization due to disruption of its disulfide bond formed with the Cys534. Amino acid residue 547–552 are involved in C-terminal looping which is part of the dimerization; therefore, Cys551Tyr could compromised the dimer formation, and disabled the active site of the protein [14]. The additional hydrogen bond formed between Tyr551 and Thr530 can affect the dipole moment of α-helix.

The novel c.761T>C (p.Leu254Ser; ClinVar# SCV000999190) allele was found in compound (*trans*) with the c.1652G>A variant in a SD patient (patient-4) whose parents were heterozygous for one of the variants. The substitution of leucine residue, a non-polar aliphatic amino acid to serine which is a polar amino acid, likely leads to altered secondary/tertiary structure of the protein and hence deleterious effect. The active sites of HEX-B enzyme compose of Arg211, Asp240, His294, Asp354, Glu355, Tyr450, Asp452, and Glu491 (Fig. 3b) which are located at (β/α)_8_-barrel structure [14]. The Leu254 is surrounded by bulky hydrophobic amino acids (Phe244, Phe246, Phe251, Trp298, Phe332, Phe336, and Phe337) and is part of the helical extension (252–259) of (β/α)_8_-barrel structure [14]. The replacement of Leu254, a hydrophobic amino acid by a more hydrophilic amino acid, Ser, could interfere the hydrophobic intermolecular contacts, leading to disruption of the cavity of the enzyme active site (Fig. 3b). Enzyme kinetic experiment or in vitro functional study is needed to confirm the pathogenic molecular mechanism of the Cys551Tyr and Leu254Ser.

The variant c.1652G>A or p.Cys551Tyr (ClinVar# VCV000167176) was first reported without clinical data in a study of SD carrier frequency for Saskatchewan in Canada of which a combined HEX-B enzyme activity and variant analysis identified a case of SD with genetic compounds between c.1652G>A and c.115delG [27]. Recently, the c.1652G>A was again described in compound heterozygous state with a nonsense variant p.Tyr463* in a Chinese infant with SD [28]. Taken together with being the common variant shared among the Thai affected infants, its absence in our control population, the highly conserved codon of Cys551, and the molecular modelling, the c.1652G>A is most likely a pathogenic allele.

Given the rarity of c.1652G>A in other populations but being the common allele among Thai patients though they were not related and were from different region of Thailand, the best plausible explanation is a founder allele. Nonetheless, we did not find evidence supporting recent common founders up to 40 generations as tested by ERSA. Why Thai and Saskatchewan affected SD patients share the intriguing allele, c.1652G>A, is difficult to answer. Saskatchewan population comprised of several native tribes and later mixed with some French descendants. Given the c.1652G>A variant does not appear to be common among Saskatchewan people, it is possible that recent migration is responsible for the presence of this allele in the population.

*HEXB* gene is the only gene known to cause SD with approximately 107 variants described with the vast majority being missense and nonsense variants [29]. Most of the *HEXB* pathologic variants are family-specific but common *HEXB* variant have been observed in certain ethnic groups such as c.850C>T (p.Arg284*) in Indian population [24], c.171delG (p.Trp57CysfsX6) in Spanish descendants [30], and c.115delG (p.Val39fs) in northern Saskatchewan population of Canada [27]. Possible genotype-phenotype correlation has been suggested, for example c.626C>T (p.Thr209Ile) and c.1404delT (p.P468PfsX62) are likely linked to the infantile form of SD [31]. Our data suggest that the p.Cys551Tyr is associated with severe infantile SD phenotypes.

Our data suggests that SD likely represents the most common subtype of infantile GM2 gangliosidosis identified among Thai patients and that TSD is much less common [7, 8]. Data on incidence/prevalence and carrier frequency of SD and TSD in Asian populations is limited, with only six Chinese [28, 31] and four Japanese cases [32] with infantile onset SD described. The carrier frequency of SD has been well demonstrated in some populations, such as 1 in 310 in Australian [33], 1 in 276 in non-Jewish American [34], and 1 in 15–27 in Saskatchewan [27]. Based on the total of 8,751,131 live births in Thailand during the study period (http://bps.moph.go.th/new_bps/healthdata), the prevalence of infantile SD is estimated at least at 1 in 1,458,521 and carrier frequency at 1 in 604 as ascertained by using Hardy-Weinberg equation. We are convinced that these numbers are far underestimated due to the lack of awareness among physicians and concomitant misdiagnosis.

The identification of the *HEXB* variants can benefit genetic counseling and prevention of reoccurrence in the affected families, as evidenced by prenatal diagnosis for the subsequent pregnancy of family-2 in the present study. Expanded carrier screening using next generation sequencing for variant detection of serious recessive disorders has been rapidly adopted by couples without existing family history of genetic conditions [35]. Having reference genome of unaffected population and disease-specific variant database of the comparable ethnic group are necessary to increase accuracy of clinically relevant interpretation of the variants identified.

## Conclusion

SD is the most common infantile onset-GM2 gangliosidosis among Thai affected population. Biochemical analysis yielded high sensitivity and at low cost, therefore it should be considered as the first diagnostic test for SD. The p.Cys551Tyr is the most common *HEXB* pathologic allele identified among Thai patients. This data is useful for designing stepwise molecular analysis and for the use of expanded carrier testing.

## Supplementary Information

**Additional file 1: Supplemental Table S1.** Primers sequences and conditions for *HEXB* mutation analysis. **Supplemental Table S2.** Scores and prediction of pathogenicity for missense variants identified using in silico analysis tools.

## Data Availability

The datasets used and/or analyzed during the current study are available from the corresponding author on reasonable request.

## Abbreviations

4MUG: 4-methylumbelliferyl-2-acetomido-2-deoxy-β-D-glucopyranoside
GM2AP: GM2 activator protein deficiency
HEX: β-hexosaminidase enzyme
HEX-A: β-hexosaminidase A
HEX-B: β-hexosaminidase B
IBD: identity-by-descent
IBS: identity-by-state
SD: Sandhoff disease
TSD: Tay-Sachs disease
