# Supplementary material for: Infantile onset Sandhoff disease: clinical manifestation and a novel common mutation in Thai patients
Source: BMC Pediatr. 2021 Jan 7;21:22. doi: 10.1186/s12887-020-02481-3 (PMC7789739; doi:10.1186/s12887-020-02481-3)
Supplement: Supplementary file 1 — Additional file 1: Supplemental Table S1. Primers sequences and conditions for HEXB mutation analysis. Supplemental Table S2. Scores and prediction of pathogenicity for missense variants identified using in silico analysis tools. [file 12887_2020_2481_MOESM1_ESM.docx]

**Supplemental Table S1** Primers sequences and conditions for *HEXB* mutation analysis

| Name of primers | Sequence (5' to 3') | | AT  (°C) | Product size (bp) |
| --- | --- | --- | --- | --- |
|  | Forward | Reverse |  |  |
| HEXb_E1 | CGCGCGCAGTCATCTGACTC | CAGTGGGGTGGTGAGGGTCT | 54 | 512 |
| HEXb_E2 | GGACTTACAATGGGCAGCAT | CTCAGTGGTTCCTAGCATGG | 64 | 355 |
| HEXb_E3 | AGGTCATGTGCTTGGGAGAA | AGGTCATGTGCTTGGGAGAA | 62 | 287 |
| HEXb_E4-5 | TTGCCTTACCTGGTTATGAGTC | ACAAATTCCCCTGTTCCAAA | 64 | 617 |
| HEXb_E6 | GGAAGCAATTCCAAATGTAGA | ACTTGTAATGAAACTATACCCA | 60 | 244 |
| HEXb_E7 | TATCAAATGCAAGCACAATTG | TATCAAATGCAAGCACAATTG | 60 | 295 |
| HEXb_E8 | TTGTAGCTTCAATAAAATGACGTAG | TCCTGACCTCAGGTGATCTGC | 64 | 400 |
| HEXb_E9 | AGTTTTTAGGCTTCTTTTTACTACC | TTGGAGTCACCATGTTACTGATTT | 64 | 400 |
| HEXb_E10 | TTGTGACACTTCCCAACTTGA | GTGGTCACTAACTACACTAGAAAGG | 56 | 351 |
| HEXb_E11 | CACATGGCACTAACTCTGAAGAA | GGTGGGAGGTTAGGGAAGAA | 60 | 440 |
| HEXb_E12-13 | TGTTGCCCTAGGATAAAGATGG | TCTATAAAACAGTCACAAGTCTGAA | 60 | 599 |
| HEXb_E14 | TCCCTTATTTTCAGTAATGCTGTG | GATGCCAGGCCTCTAAATGT | 56 | 350 |

Reference sequences: NT_006713, NM_000521

**Supplemental Table S2**  Scores and prediction of pathogenicity for missense variants identified using *in silico* analysis tools

| Analysis tools | Scores/ Prediction of pathogenicity | |
| --- | --- | --- |
|  | c.1652G>A (p.Cys551Tyr) | c.761T>C (p.Leu254Ser) |
| SIFT 5.1.1 | 0/ damaging | 0/ damaging |
| PolyPhen2 | 1.000/ probably damaging | 1.000/ probably damaging |
| PROVEAN | -9.773/deleterious | -5.846/ deleterious |
| PredictSNP2 | 1.0000/ deleterious | 1.0000/ deleterious |
| CADD^a^ | 21/ deleterious | 24.6000/ deleterious |
| DANN^b^ | 0.9967/ deleterious | 0.9941/ deleterious |
| FATHMM^c^ | 0.9951/ deleterious | 0.9905/ deleterious |
| FunSeq2 | 3.0000/ deleterious | 3.000/ deleterious |
| GWAVA^d^ | 0.5900/ deleterious | 0.6500/ deleterious |

^a^ CADD, Combined Annotation Dependent Depletion

^b^ DANN, Deleterious Annotation of genetic Variants using Neural Networks

^c^ FATHMM, Functional Analysis through Hidden Markov Models

^d^ GWAVA, Genome-wide Annotation of Variants
